# Characterisation of the complete chloroplast genome of *Lycoris longituba* (Amaryllidaceae)

**DOI:** 10.1080/23802359.2019.1681324

**Published:** 2019-10-25

**Authors:** Fengjiao Zhang, Haiying Tong, Hong Yang, Tao Wang, Weibing Zhuang, Xiaochun Shu, Zhong Wang

**Affiliations:** Jiangsu Key Laboratory for the Research and Utilization of Plant Resources, Institute of Botany, Jiangsu Province and Chinese Academy of Sciences (Nanjing Botanical Garden Mem. Sun Yat-Sen), Nanjing, China

**Keywords:** *Lycoris longituba*, complete chloroplast genome, phylogenetic analysis

## Abstract

*Lycoris longituba* is a species in the Amaryllidaceae with high ornamental and medicinal value. It is also an endangered species in East China due to the narrow distribution. Here, we assembled and characterised the complete chloroplast (cp) genome of this species using high throughput sequencing and bioinformatics analysis. As a result, the whole cp genome size is 158,484 bp, including two inverted repeat (IR) regions of 26,765 bp, large single-copy region (LSC) of 86,458 bp and small single-copy region (SSC) of 18,496 bp. A total of 137 genes were identified, including 87 protein-coding genes, 42 tRNA genes, and eight rRNA genes. Phylogenomic analysis was carried out using complete cp genome of 18 species in five families, supporting the closer relationship between *L. longituba* and *L. squamigera* than *L. radiata* and *L. sprengeri* in Amaryllidaceae.

*Lycoris longituba* is native to China and distributed in East China (He et al. 2011). With the characteristics of large and colourful flowers, it was planted as an ornamental plant (He et al. 2010). However, due to the similar flower with other species and many variants, it is not easy to identify and classify by morphology. Chloroplast genome sequences are efficient DNA molecular markers for species identification and phylogenetic relationships in plants (Freitas et al. 2018). Here, we assembled and characterised the complete chloroplast of *L. longituba* using Illumina sequencing and bioinformatics analysis, which will provide more information for the molecular phylogeny construction and classification in the genus *Lycoris*.

*Lycoris longituba* bulbs were planted in Nanjing Botanical Garden, Mem. Sun Yat-sen (E118_83, N32_06), Nanjing, China. Specimens (no. NAS00585500) was stored at herbarium of Institute of Botany, Jiangsu Province and Chinese Academy of Science. Fresh leaves were collected in spring and stored in liquid nitrogen for DNA extraction. DNA was extracted using the plant DNA isolation reagent (Code: D9194, TaKaRa, China) according to the instructions. After the detection of DNA purity and integrity, qualified DNA was used to library construction and sequence on Illumina Noveseq at Novogene company (http://www.novogene.com/). A total of 199 million clean reads (paired-end 150) were generated, and 39 million reads were used to chloroplast genome assembly using the organelle assembler NOVOPlasty Version 3.3 (Dierckxsens et al. 2017). *Lycoris squamigera* cp genome (GenBank accession MH118290.1) (Jin et al. 2018) was used as reference sequence. Finally, genome annotation, visualisation, and tandem repeats identification were completed on web server CPGAVAS2 (http://www.herbalgenomics.org/cpgavas2) (Shi et al. 2019). The complete chloroplast genome was deposited in GenBank (accession no. MN096601).

The chloroplast genome of *L. longituba* was 158,484 bp with 37.8% GC content, including a large single-copy (LSC) region of 86,458 bp, a small single-copy (SSC) region of 18,496 bp and two equal length inverted repeat (IR) regions of 26,765 bp. A total of 137 genes were predicted and annotated, which contains 87 protein-coding genes, 42 tRNA genes, and eight rRNA genes. Of these, 17 genes were splitting genes with introns and exons, including 15 genes with a single intron and two genes (*ycf3* and *clpP*) with two introns. In *L. radiata* (Zhang et al. 2019) and *L. sprengeri* (Zhang et al. 2019), there was one more gene *ndhF* with a single intron.

Phylogenetic analysis was performed between *L. longituba* and other related taxa, including 18 species in five families. The complete cp genome sequences were downloaded from the NCBI GenBank database and aligned using MAFFT (version 7) online service with default parameters (https://mafft.cbrc.jp/alignment/server/) (Rozewicki et al. 2019). A phylogenetic tree was constructed using neighbour-joining (NJ) phylogeny with the Jukes–Cantor model and 1000 bootstrap replicates according to the manual on MAFFT online service (Figure 1). The result showed that *L. longituba* was grouped together with *L. squamigera* but on the sub-branches with *L. radiata* and *L. sprengeri* in *Lycoris*. These four species of *Lycois* were closely related to *Narcissus* and *Allium* in the Amaryllidaceae.

**Figure 1.**
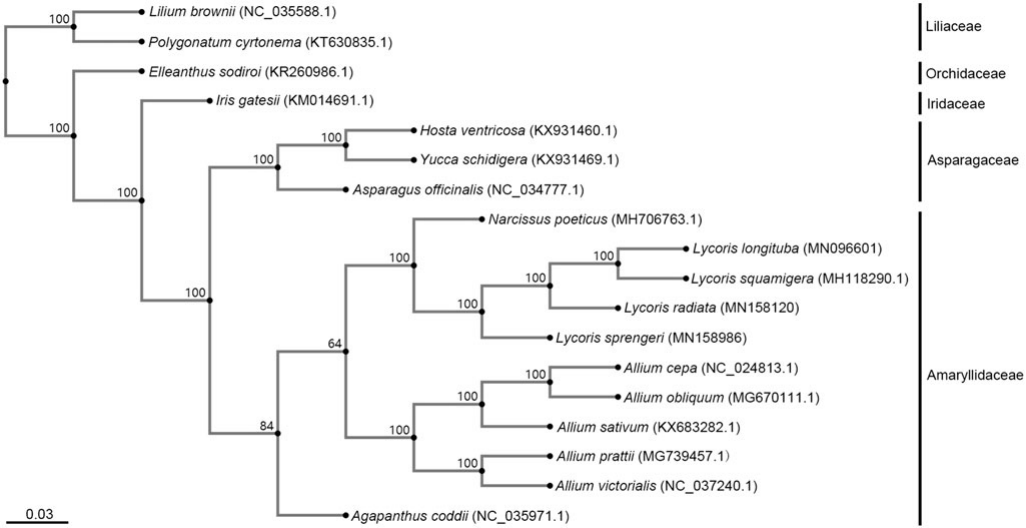
Phylogenetic relationships based on the cp genome sequences of 18 species, showing the phylogenetic position of *L. longituba* in *Lycoris*, which is closer with the *L. squamigera*. The bootstrap values based on 1000 replicates were shown on the nodes, the names and Genbank accession number of the species were shown at the end of each branch.
